# Community Violence Exposure and Conduct Problems in Children and Adolescents with Conduct Disorder and Healthy Controls

**DOI:** 10.3389/fnbeh.2017.00219

**Published:** 2017-11-06

**Authors:** Linda Kersten, Noortje Vriends, Martin Steppan, Nora M. Raschle, Martin Praetzlich, Helena Oldenhof, Robert Vermeiren, Lucres Jansen, Katharina Ackermann, Anka Bernhard, Anne Martinelli, Karen Gonzalez-Madruga, Ignazio Puzzo, Amy Wells, Jack C. Rogers, Roberta Clanton, Rosalind H. Baker, Liam Grisley, Sarah Baumann, Malou Gundlach, Gregor Kohls, Miguel A. Gonzalez-Torres, Eva Sesma-Pardo, Roberta Dochnal, Helen Lazaratou, Zacharias Kalogerakis, Aitana Bigorra Gualba, Areti Smaragdi, Réka Siklósi, Dimitris Dikeos, Amaia Hervás, Aranzazu Fernández-Rivas, Stephane A. De Brito, Kerstin Konrad, Beate Herpertz-Dahlmann, Graeme Fairchild, Christine M. Freitag, Arne Popma, Meinhard Kieser, Christina Stadler

**Affiliations:** ^1^Department of Child and Adolescent Psychiatry, Psychiatric University Hospital, University of Basel, Basel, Switzerland; ^2^Department of Child and Adolescent Psychiatry, VU University Medical Center, Amsterdam, Netherlands; ^3^Department of Child and Adolescent Psychiatry, Psychosomatics and Psychotherapy, University Hospital Frankfurt, Goethe University, Frankfurt am Main, Germany; ^4^Department of Psychology, University of Southampton, Southampton, United Kingdom; ^5^Broadmoor High Secure Hospital, West London Mental Health NHS Trust, Crowthorne, United Kingdom; ^6^School of Psychology, University of Birmingham, Birmingham, United Kingdom; ^7^Department of Child and Adolescent Psychiatry, Psychosomatics and Psychotherapy, University Hospital, RWTH Aachen, Aachen, Germany; ^8^Basurto University Hospital, Bilbao, Spain; ^9^Faculty of Medicine, Child and Adolescent Psychiatry, Department of the Child Health Center, Szeged University, Szeged, Hungary; ^10^Department of Child and Adolescent Psychiatry, National and Kapodistrian University of Athens, Athens, Greece; ^11^University Hospital Mutua Terrassa, Barcelona, Spain; ^12^Center of Addiction and Mental Health, Toronto, ON, Canada; ^13^Department of Psychiatry, Medical School, National and Kapodistrian University of Athens, Athens, Greece; ^14^Department of Psychology, University of Bath, Bath, United Kingdom; ^15^Institute of Medical Biometry and Informatics, University of Heidelberg, Heidelberg, Germany

**Keywords:** community violence exposure, conduct disorder, reactive aggression, proactive aggression, adolescence, antisocial behavior

## Abstract

Exposure to community violence through witnessing or being directly victimized has been associated with conduct problems in a range of studies. However, the relationship between community violence exposure (CVE) and conduct problems has never been studied separately in healthy individuals and individuals with conduct disorder (CD). Therefore, it is not clear whether the association between CVE and conduct problems is due to confounding factors, because those with high conduct problems also tend to live in more violent neighborhoods, i.e., an ecological fallacy. Hence, the aim of the present study was: (1) to investigate whether the association between recent CVE and current conduct problems holds true for healthy controls as well as adolescents with a diagnosis of CD; (2) to examine whether the association is stable in both groups when including effects of aggression subtypes (proactive/reactive aggression), age, gender, site and socioeconomic status (SES); and (3) to test whether proactive or reactive aggression mediate the link between CVE and conduct problems. Data from 1178 children and adolescents (62% female; 44% CD) aged between 9 years and 18 years from seven European countries were analyzed. Conduct problems were assessed using the Kiddie-Schedule of Affective Disorders and Schizophrenia diagnostic interview. Information about CVE and aggression subtypes was obtained using self-report questionnaires (Social and Health Assessment and Reactive-Proactive aggression Questionnaire (RPQ), respectively). The association between witnessing community violence and conduct problems was significant in both groups (adolescents with CD and healthy controls). The association was also stable after examining the mediating effects of aggression subtypes while including moderating effects of age, gender and SES and controlling for effects of site in both groups. There were no clear differences between the groups in the strength of the association between witnessing violence and conduct problems. However, we found evidence for a ceiling effect, i.e., individuals with very high levels of conduct problems could not show a further increase if exposed to CVE and vice versa. Results indicate that there was no evidence for an ecological fallacy being the primary cause of the association, i.e., CVE must be considered a valid risk factor in the etiology of CD.

## Introduction

Community violence exposure (CVE) is defined as the witnessing of violence within a community, falling victim to violent acts oneself, or being subjected to a combination of both experiences (Schwab-Stone et al., 1995). CVE is a common and persistent public health issue in many inner city neighborhoods (Buka et al., 2001; Stein et al., 2003). Although the prevalence of CVE has been reported to be lower in many European countries compared to the US (Mercy et al., 2003; Hillis et al., 2016), it has nevertheless been recognized as a global public health problem by the World Health Organization (2002). A comprehensive meta-analysis of 114 studies on the effects of CVE on adolescent mental health by Fowler et al. (2009) found that the effects of CVE were strongest on the subsequent development of post-traumatic stress disorder, followed closely by externalizing problems. Specifically, effect sizes for the relationship between CVE and externalizing problems were 0.72–0.78 for witnessing violence and victimization, respectively. Fowler et al. (2009) found that relationship between exposure to CVE and externalizing behaviors was stronger in adolescents compared to children. Further factors that have been shown to influence the effects of CVE on mental health outcomes are gender and socioeconomic status (SES). Namely, being male (Javdani et al., 2014), and coming from lower SES strata (Anderson et al., 2001) have been found to increase the strength of the association between CVE and conduct problems.

Overall, research evidence to date suggests that the association of CVE and conduct problems is reciprocal, enhancing the chances of a negative spiral of increasing conduct problems and greater violence exposure. For instance, a bi-directional relationship between CVE and externalizing problems has been reported by Mrug and Windle (2009). The authors found that CVE was linked to the development of later conduct problems and delinquency. Likewise, baseline delinquency predicted higher rates of later CVE. Although there is much evidence indicating that violence exposure in early childhood is a major risk factor for the development of Conduct Disorder (CD; for a review see Burke et al., 2002), to date it is not known whether there are similarly strong associations between recent CVE and current conduct problems in adolescents with CD. If a reciprocal relationship between CVE and conduct problems exists, strong associations between recent CVE and current conduct problems would be expected.

Children and adolescents with CD constitute a group that is *particularly* prone to experiencing violence exposure due to the nature of their diagnosis. CD is defined as a repetitive and persistent pattern of violent and antisocial behavior (American Psychiatric Association, 2013). For children and adolescents with CD, it is difficult to separate CVE as a form of unintended environmental exposure from self-provoked situations that reflect part of the adolescent’s symptomatology (Halliday-Boykins and Graham, 2001; Lynch, 2003). Children and adolescents with CD may encounter violent situations in ways other than as innocent bystanders, e.g., as a result of being present when a friend initiates a fight or robs a person or indeed as the perpetrator themselves. Much of the CVE literature focuses on community samples derived from urban, low socio-economic backgrounds, representing ethnic minorities and living in neighborhoods with high crime rates (Dempsey et al., 2000; Gorman-Smith et al., 2004; Frey et al., 2009; Copeland-Linder et al., 2010; Goldner et al., 2015). As such, these studies have likely included a mixture of healthy and clinically impaired youth. According to epidemiological research around 22.2% of adolescents within a national representative US sample reported a history of a psychiatric disorder that was accompanied by severe impairment or distress, of which 9.6% comprise behavioral disorders, such as CD or attention-deficit/hyperactivity disorder (Merikangas et al., 2010). For European countries specifically, it is estimated that around 38.2% of the general population of the European Union (EU) exhibit a mental disorder each year, with 5% of that proportion relating to externalizing behavior (Wittchen et al., 2011). Generally, adolescents from low-income neighborhoods exhibit greater mental health problems than those living in higher-income neighborhoods (Aneshensel and Sucoff, 1996). Although past CVE studies have offered unique insights into the debilitating effects of CVE on adolescents’ psychosocial adjustment, the effects of CVE remain to be disentangled among a sample in which healthy and clinically-impaired individuals can be distinguished. Investigating these two groups separately allows precluding the presence of an ecological fallacy, i.e., the finding of stronger associations between CVE and conduct problems than is actually the case, due to the aggregation of healthy and clinically impaired adolescents. Specifically, insight into the associations between recent CVE and current conduct problems in an adolescent sample with CD and a healthy control sample will answer the following questions: does recent CVE continue to be of relevance in terms of determining current conduct problems in healthy adolescents as well as in those who have developed diagnosable levels of conduct problems, i.e., those with a CD diagnosis? Can we exclude an ecological fallacy that may have developed due to a lack of studies investigating the effects of CVE and conduct problems in an exclusive group of healthy adolescents vs. adolescents with CD?

Studies have shown that effects of CVE on later conduct problems persisted even when controlling for an individual’s initial aggression level (e.g., Schwab-Stone et al., 1995; Farrell and Bruce, 1997; Miller et al., 1999; McCabe et al., 2005; Weaver et al., 2008). However, aggression is heterogeneous and may take different forms. Two key forms of aggression that are commonly distinguished are reactive and proactive aggression (for overview, see Kempes et al., 2005). Reactive aggression refers to impulsive forms of aggression, usually evoked by high arousal levels and strong emotions such as anger or fear. In contrast, proactive aggression is an instrumental, often pre-meditated form of aggression, characterized as callous and goal-oriented behavior and thought to be associated with low levels of arousal. Reactive aggression may be explained through the frustration-anger model (Dollard et al., 1939), explaining why this form of aggression is commonly linked to provocations or threats. Proactive aggression, on the other hand, is better understood through social learning theory (Bandura, 1973). This theory outlines why proactive aggression is often motivated by reward-orientation and is reinforced by positive outcomes following aggressive behavior. There have been more recent theories proposed since then which have set out hypotheses regarding the distinct neurobiological bases of these two aggression subtypes. For instance, reactive aggression has been linked to orbitofrontal cortex dysfunction and impaired emotion regulation (Bechara et al., 2000; Blair and Cipolotti, 2000) while proactive aggression is thought to be associated with amygdala dysfunction and a diminished response to distress cues (Blair, 1995, 2005). Research has shown that proactive but not reactive aggression may be predictive of later delinquency, conduct problems and violent offending in mid-adolescence as well as criminal behavior later in life (Pulkkinen, 1996; Vitaro et al., 1998; Raine et al., 2006). Conversely, reactive aggression was found to predict impulsivity and hostility (Raine et al., 2006).

Thus, when investigating the association between CVE and conduct problems, it is not only necessary to parse out effects of aggression but also to examine the role of each of these aggression subtypes separately. To date, it remains to be investigated how the relationship between recent CVE and current conduct problems may differ according to aggression subtypes. One possibility is that greater violence exposure is associated with more proactive aggression, perhaps because such exposure normalizes violence or leads to a desensitization to the effects of violence. More proactively aggressive children and adolescents, in turn, may intentionally choose to enter violent situations. Another possibility is that greater violence exposure is associated with more reactive aggression, possibly due to its effects on sensitivity to threat or even the neural circuits implicated in reactive aggression. Individuals with high levels of reactive aggression may, in turn, act out aggressively in response to CVE.

In summary, many studies have shown that there is a strong association between CVE and conduct problems. However, to date no study has investigated this association separately in children and adolescents with a diagnosis of CD and a sample exclusively made up of healthy controls to examine the deleterious effects of CVE separately in high and low-risk groups. Finally, the literature has not differentiated between reactive and proactive forms of aggression in terms of possible mediators of the association between CVE and conduct problems. We know that the relationship between CVE and aggression still holds when controlling for levels of prior aggression. Understanding how different types of aggression (i.e., reactive vs. proactive) may explain the link between CVE and conduct problems within healthy controls vs. children and adolescents with CD might be important for further specifying etiological models.

We had the following hypotheses:

1. We expected to observe a strong association between recent CVE and current conduct problems in children and adolescents with CD as well as in healthy controls.
2. This relationship between CVE and current conduct problems was expected to hold, even when accounting for effects of aggression subtypes.
3. We tested (exploratively) whether the association between CVE and conduct problems is primarily mediated by proactive or reactive aggression in children and adolescents with CD and healthy controls.
4. We expected increased age, lower SES and male gender to be linked to greater rates of CVE as well as conduct problems in both groups.

## Materials and Methods

This study was conducted within the framework of the ongoing European multi-disciplinary FP7 (i.e., European Commission’s 7th Framework Health program) project “Neurobiology and Treatment of Adolescent Female Conduct Disorder: The Central Role of Emotion Processing” (FemNAT-CD). A detailed outline of the methodological aspects of the project is available on the official website^1^. Assessments were conducted at clinical sites from seven European countries: Germany, Greece, Hungary, Netherlands, Spain, Switzerland and the UK.

### Participants

Child and adolescent participants between the ages of 9 and 18 years were recruited through various means, including distribution of study information in schools, sports and leisure clubs, through street promotion and contacts with psychiatric clinics, youth offending services, or youth welfare institutions.

The inclusion criterion for the CD sample was a current diagnosis of CD according to DSM-IV-TR criteria (American Psychiatric Association, 2000). Exclusion criteria for both CD and control groups were a history and/or current diagnosis of autism spectrum disorder, schizophrenia, bipolar disorder or mania, fetal alcohol syndrome (all according to DSM-IV-TR), any known monogenetic disorders, chronic or acute neurological disorders, severe medical conditions or valid indications of an IQ < 70 (measured with the vocabulary and block design subtests of the Wechsler Intelligence Scale for Children (WISC) or vocabulary and matrix reasoning subtests of the Wechsler Adult Intelligence Scale (WAIS; Wechsler, 2008) depending on the participant’s age; at UK sites, the Wechsler Abbreviated Scale of Intelligence was used for all ages (Wechsler, 1999). Additional exclusion criteria for healthy controls included any other current disorder according to DSM-IV-TR criteria as well as a past history of Attention-Deficit/Hyperactivity Disorder, Oppositional Defiant Disorder or CD.

From the current study sample, 1178 children and adolescents had complete data on all key measures and thus were included in the present analysis. Consistent with the aim of the study to over-recruit female participants, there were more females than males (62 vs. 38%) and slightly more than half of the overall sample was healthy controls (56%). The number of male children and adolescents was spread evenly across CD and control subjects (50% each). With regard to females, there were slightly more controls than CD subjects (60 vs. 40%). Comparison of the CD and control groups suggested that children and adolescents with CD were significantly older than controls (*M* age CD = 14.4, *SD* = 2.3 vs. *M* age controls = 13.9, *SD* = 2.6, *t* = 3.63, *p* < 0.001) and were characterized by significantly lower SES (*M SES* CD = −0.3, *SD* = 0.9 vs. *M* SES controls = 0.3, *SD* = 1.0, *t* = −9.36, *p* < 0.001).

### Procedure

Participants and their legal guardians received detailed study information via telephone, mail or email prior to the day of assessment. On the first assessment day, participants were given the opportunity to ask questions and it was assured that both parent/legal guardian and children knew that participation could be declined or stopped at any point during the course of the study. Written informed consent was obtained from the participants and their legal guardians. If consent of the legal guardian was unavailable, participants were included only if considered old enough according to the ethical requirements of the respective country (i.e., ≥16 in Switzerland and UK, ≥18 all else). Research was carried out in compliance with the fundamental ethical principles as stated by the Declaration of Helsinki and its later amendments as well as with the ethical standards of the institutional and/or national research committee. All subjects and legal guardians gave written informed consent in accordance with the Declaration of Helsinki. If it was not possible to obtain consent from the legal guardian, participants were included only if considered old enough to provide informed consent according to the ethical requirements of the respective country (i.e., <=16 years in Switzerland and the UK, <=18 years in all other countries). Ethical approval was obtained from all local ethics committees (Basel—Ethikkommission Nordwest- und Zentralschweiz, Frankfurt—Ethik-Kommission Fachbereich Medizin Klinikum der Johann Wolfgang Goethe-Universität, Aachen—Ethik-Kommission an der medizinischen Fakultät der rheinisch-westfälischen technischen Hochschule Aachen, Amsterdam—Medisch Ethische Toetsingscommissie Vrije Universiteit Medisch Centrum, Birmingham and Southampton—NHS Research Ethics Committee, Bilbao—Comite Etico de Investigacion Hospital Universitario Basurto, Barcelona—Comite Etico de Investigacion Clinica Parc de Salut Mar, Szeged—Human Reproduction Committee, Athens—Ethics Committee of Aiginiteio University Hospital of Athens).

In order to obtain information on mental health problems, semi-structured interviews were conducted with the children/adolescents and, if available, with their legal guardian in separate rooms/consecutively by trained, postgraduate-level investigators. Information obtained from both interviews was then combined to obtain a final summary judgment. Questionnaires assessing CVE were handed to the participant subsequent to the interview. Investigators were available to provide help to participants and clarify the meaning of items if requested. In line with the ethics committees’ decision for the respective universities, participants were compensated with a gift card or a small monetary payment.

### Measures

#### Community Violence Exposure

Initially developed by Richters and Saltzman (1990), and modified by Schwab-Stone et al. (1995, 1999) and Ruchkin et al. (2004), two scales of the Social And Health Assessment (Weissberg et al., 1991) assessing direct victimization as well as the witnessing of violence in the community, served as the measure of CVE. For the victimization scale, seven items assessed how often in the past year participants had been: (1) beaten up or mugged; (2) threatened with serious physical harm by someone; (3) threatened because of their race/ethnicity; (4) shot or shot at with a gun; (5) attacked or stabbed with a knife; (6) chased by gangs or individuals; or (7) seriously wounded in an incident of violence. Participants reported their answer on a 5-point Likert scale from 0 (never), 1 (1–2 times), 2 (3–5 times), 3 (6–9 times) to 4 (10 times or more). The witnessing scale included seven items asking the respondents how frequently they had seen someone else being exposed to the same violent acts in their community within the past year on the same 5-point Likert scale as described above. The two scales were found to have good psychometric properties in a sample of American inner-city youth (Richters and Martinez, 1993). With respect to our sample, the witnessing subscale produced a Cronbach’s alpha of 0.89 for cases and 0.77 for controls, i.e., good/satisfactory internal consistency respectively. The victimization subscale resulted in an alpha of 0.81 for cases and 0.67 for controls indicating good/questionable internal validity (Bland and Altman, 1997).

#### Conduct Problems

The Kiddie Schedule for Affective Disorders and Schizophrenia—Present and Lifetime (K-SADS-PL; Kaufman et al., 1997) is a semi-structured diagnostic interview used to screen for the current presence or lifetime history of a broad range of disorders ranging from affective disorders (i.e., depression, bipolar disorder), schizophrenia and substance use disorders through to externalizing disorders (i.e., CD, ADHD). The K-SADS-PL is administered independently to the adolescent as well as their caretaker to assess the presence of DSM-IV-TR psychiatric disorders (for this study, the CD present section was used). Summary ratings are derived from clinical judgment using both interview sources as well as other information available on file. The items of the instrument are scored on a scale from 0 to 3. A rating of 0 indicates no (insufficient) information, a score of 1 indicates a given symptom is not present, 2 indicates a subclinical expression, while a score of 3 is given when a symptom is present and clinically significant. Scores were recoded, so that a clinical rating of “not present” is represented by 0, a subclinical rating by a score of 1, and a clinically significant rating by a score of 2. Unknown ratings were recoded into missing data. For the purpose of the current study, mean item scores were calculated for CD based on the current summary ratings. That is, a mean score was calculated across all CD symptoms for each individual. This procedure allowed inspection of current conduct problems at the *symptom-level* and therefore represented a more comprehensive estimation of problematic behavior symptoms with regard to the healthy control group. In other words, conduct problems were assessed on a dimensional level including subclinical expressions to assess the association between CVE and conduct problems. Inter-rater reliability for the K-SADS-PL section used was based on 75 CD individuals and found to be almost perfect with a percentage agreement of 94.67 and Cohen’s κ of 0.907 (95% *CI*: 0.819–0.995; Landis and Koch, 1977).

#### Aggression Subtypes

Developed by Raine et al. (2006), the Reactive-Proactive aggression Questionnaire (RPQ) measures self-reported reactive (11 items, e.g., “I have damaged things because I felt mad”, “I have gotten angry when frustrated”, “I have had temper tantrums”) and proactive (12 items, e.g., “I have had fights to show that I was on top”, “I have vandalized something for fun”, “I have gotten others to gang up on someone”) aggression. Each item is answered on a 3-point Likert scale (0 = never, 1 = sometimes, 2 = often). The two scales were found to have good internal validity (0.84 for reactive, 0.86 for proactive aggression) in its original evaluation study comprising a sample of American school boys (Raine et al., 2006). Further validation studies in several countries have subsequently confirmed reliability and validity across the genders and various populations (e.g., different age groups, non-offender vs. criminal samples; Fossati et al., 2009; Fung et al., 2009; Cima et al., 2013). With regard to the present sample, Cronbach’s alpha was 0.84 for adolescents with CD and 0.79 for controls for the reactive aggression subscale indicating good to satisfactory internal consistency. The proactive subscale yielded estimates of 0.85 for adolescents with CD and 0.67 for controls indicating good/questionable internal consistency (Bland and Altman, 1997).

#### Socioeconomic Status

SES was calculated based on parental income, education as well as occupational status. Classifications were made using the International Classification of Education (UNESCO Institute for Statistics, 2015) and the International Standard Classification of Occupations (International Labour Organization, 2012). Human rater and computer-based ratings were combined into a standardized factor (*M* = 0, *SD* = 1) score using Principal Component Analysis. Internal consistency of the composite SES score was acceptable (α = 0.74). Due to potential economic variation on the country level, SES was centered and scaled within each country, in order to obtain an indicator of relative socioeconomic position.

### Data Analysis

The data were analyzed using the Statistical Package for Social Sciences (SPSS-23; IBM Corp, 2016, Armonk, NY, USA), Analysis of Moment Structures (AMOS-23; Arbuckle, 2014) and R (R Core Team, 2013) with the packages “plot3D”, “ggplot2” and “localgauss”. For descriptive results sample mean scores were calculated for witnessing, victimization, conduct problems, reactive and proactive aggression measures to characterize the two groups. Furthermore, Mann-Whitney-U tests and two-sample sample *t*-tests were calculated to gain more insight into group differences. Local Gaussian correlations and 2-D plots were computed to approximate density functions and obtain further insight into the distribution of CVE and conduct problems within the two groups. Structural equation modeling (SEM) was used for analyzing the primary models since it allowed us to compare the model fit of successively nested models with each other. In all SEM models age, gender, site and SES were used as control variables. For the final model age, gender and SES were inspected as moderating variables, while site served as a control variable.

Analyses were conducted on two different CVE constructs: (1) witnessing violence was examined as a latent variable by parceling the seven items comprising this scale into three indicators; (2) using the same procedure, victimization was inspected separately as well. Through the use of latent variables, we were able to reduce measurement errors and improve the accuracy of the findings (Little et al., 1999). The method of parceling was chosen in order to overcome low communality and reliability frequently encountered with the use of individual items and to decrease the likelihood for distributional violations (Little et al., 1999, 2002). Items were grouped into parcels based on item-total correlations (Little et al., 1999, 2002). Items with highest and lowest item-total correlations were grouped together, resulting in two groups with two items and one group with three items (see Table 1 for detailed list of parcel composition). The parceled indicators loaded well on their respective factors with loadings ranging between 0.59 and 0.88 (see Table 1).

**Table 1:** Parcel composition and standardized loadings of parceled indicators by group.

	Witnessing items	Victimization items	Witnessing loading (CD/Control)	Victimization loading (CD/Control)
Parcel 1	Beat up	Beat up	0.88/0.65	0.85/0.68
	Gun shot	Gun shot		
	Discrimination	Discrimination		
Parcel 2	Chasing	Chasing	0.84/0.88	0.75/0.63
	Threats	Knife attack		
Parcel 3	Knife attack	Threats	0.85/0.87	0.81/0.59
	Serious wound	Serious wound		

Chi-square, the Root Mean Square Error of Approximation (RMSEA; Browne and Cudeck, 1993) and the Comparative Fit Index (CFI; Bentler, 1990) were used as indicators of goodness of fit. While for the commonly employed Chi-Square test greater (insignificant) *p*-values generally indicate better fit, the RMSEA requires values of 0.05 or less and CFI values of 0.95 or greater to consider a model to be of acceptable fit (Bentler, 1990; Browne and Cudeck, 1993).

To test the mediation hypotheses, change in model fit when direct paths from CVE to conduct problems were removed was assessed controlling for site and moderating effects of age, SES and gender (Holmbeck, 1997). Additionally, the magnitude of the indirect effects of CVE on conduct problems via reactive or proactive aggression was estimated (Holmbeck, 1997). For the group comparison, a series of up to four models were examined for cases and controls separately, including comparisons between an exploratory model in which all paths between the variables were free to vary for each group. This model served to pinpoint the variables of interest for each group. Then, a fully constrained model was examined, in which all primary paths were set as equal for both groups. Consequently, the second model hypothesized no group differences for all associations/paths. If this model was true, constraining the paths to the same value should not significantly decrease the overall model fit (as compared to the first model). If indicated (i.e., if second model significantly decreased model fit) a third model, in which some selected paths were non-constrained, was inspected. These selected paths were identified by re-examining the paths of the first model and selecting potentially different associations between patients and controls. The selected paths were unconstrained and allowed to differ by group. If the model fit significantly improved compared to the second model (and was not worse than that for the first model), it would suggest the presence of group differences in the model. A final select model would then be produced in which all insignificant paths are deleted and again compared against the model fit of the previous model.

## Results

### Descriptive Analyses

**Figure 1:**
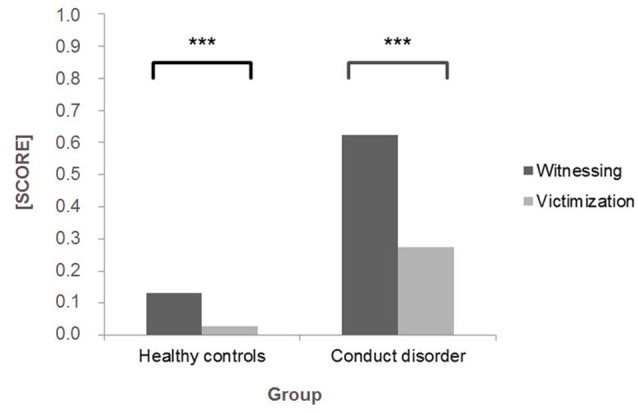
Mean scores (with a possible range of 0–4) for SAHA witnessing violence and victimization subscales within the past year reported by healthy controls (*n* = 662) and children and adolescents with conduct disorder (CD; *n* = 516). ***p < 0.001.

Children and adolescents with CD reported significantly greater CVE within the past year than healthy controls for both witnessing violence, *U* = 88840, *p* < 0.001 (*M* witnessing CD = 0.62, *SD* = 0.75 vs. *M* witnessing controls = 0.13, *SD* = 0.29) and victimization, *U* = 97557, *p* < 0.001 (*M* victimization CD = 0.28, *SD* = 0.46 vs. *M* victimization controls = 0.03, *SD* = 0.11; see Figure 1). As healthy controls rarely reported victimization events within the past year, only the witnessing violence subscale of the SAHA was included in all further analyses. In both groups the distribution of CVE was skewed as many individuals reported zero to low frequency of exposure within the past year. Table 2 presents the means and prevalence rates (i.e., the percentage of individuals having experienced the respective item at least once within the past year) of each witnessing item by group and shows that children and adolescents with CD experienced all of the listed events to a much greater extent than their healthy counterparts. Supplementary Table S1 (presented in Supplementary Material) shows the exact percentage of endorsed frequencies within the past year by group. In both groups, “threats with physical harm” was the most frequently endorsed form of violence exposure (49.5% vs. 16.0%), while “getting shot” was the least frequently encountered event (12.2% vs. 0.9%) by children and adolescents with CD and healthy controls, respectively. In addition, means and standard deviations for reactive and proactive aggression are presented in Table 2.

**Table 2:** SAHA witnessing subscale items and reactive and proactive aggression questionnaire mean scores by group.

Witnessing item	*CD* (*N* = 516) Mean (% endorsed yes)	*Controls* (*N* = 662) Mean (% endorsed yes)
*In the past year, have you seen someone else…*		
1. Being chased by gangs/individuals	0.73 (45.0)	0.16 (12.6)
2. Get threatened with serious physical harm	0.99 (55.1)	0.21 (16.4)
3. Getting beaten up/mugged	0.84 (47.4)	0.18 (14.0)
4. Being attacked/stabbed with a knife	0.43 (28.6)	0.05 (4.4)
5. Seriously wounded after an incident of violence	0.60 (38.0)	0.12 (9.6)
6. Getting shot/shot at with a gun	0.18 (13.6)	0.02 (0.9)
7. Getting threatened/harmed for their race/ethnicity	0.61 (34.3)	0.16 (12.5)
Reactive aggression	1.07 (0.46^1^)	0.50 (0.33^1^)
Proactive aggression	0.38 (0.38^1^)	0.10 (0.13^1^)

*^1^Values represent corresponding standard deviations*.

**Figure 2:**
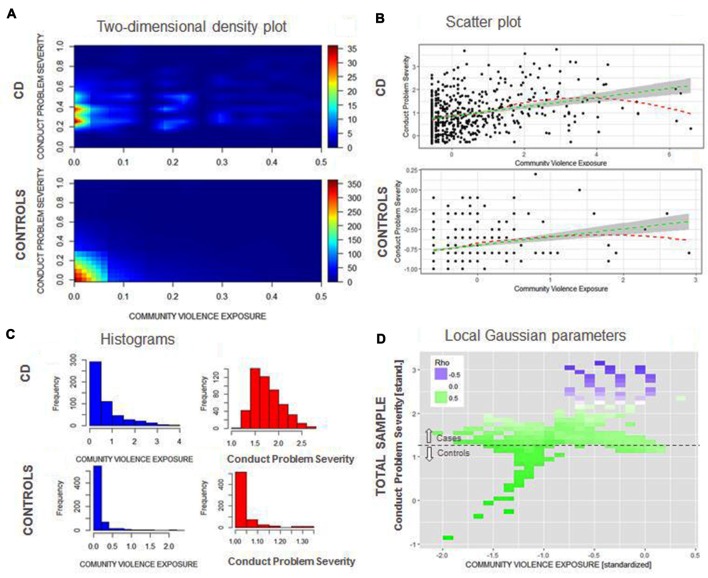
**(A)** Two-dimensional density plot for community violence exposure (CVE; *x*-axis) and current conduct problems (*y*-axis) for adolescents with CD (top) and healthy controls (bottom) using plot3D. **(B)** Scatterplot and linear (green) and loess (red) regression line for CVE (*x*-axis) and conduct problems (*y*-axis) for adolescents with CD (top) and controls (bottom) ggplot2. **(C)** Histograms for both CVE and current conduct problems for adolescents with CD and controls. **(D)** Stability of the correlation between CVE and current conduct problems along the range of both variables (high correlations = green; low correlations = purple) using {localgauss}.

Figures 2A,C shows a broader range of witnessed violence for adolescents with CD than for healthy controls, although high values for witnessing are rare amongst both groups. For controls, recent witnessing and current conduct problems have a highly left-skewed distribution, since most individuals have low current conduct problems and low frequency of recent exposure to witnessed violence. For adolescents with CD, current conduct problems are more normally distributed (Figure 2C). Figure 2B shows that for both adolescents with CD and healthy controls a positive linear trend (green line) between recent witnessing and current conduct problems can be observed. Testing the significance of the association of CVE and conduct problems between groups in a SEM model, while controlling for site effects as well as the moderating effects of age, gender and SES indeed revealed significant associations for both groups (CD: beta = 0.36, *p* < 0.001; and controls: beta = 0.20, *p* < 0.001). A Loess fitting function (red line) shows that the fit line flattens or even becomes slightly negative in the higher range of current conduct problems or witnessing violence. This finding can be corroborated assessing local Gaussian parameters (Figure 2D): Local Gaussian parameters show a positive trend across the whole spectrum of current conduct problems/witnessing violence, whereas in the higher range of both variables, the association becomes neutral (white) or even negative (purple). These findings indicate a “ceiling” effect, i.e., that beyond a high level of current conduct problems, witnessing violence is not able to increase the level of symptoms, and vice versa.

### Mediation Analysis of Reactive and Proactive Aggression in the Overall Sample

Reactive and proactive aggression partially mediated the relationship between witnessing community violence and conduct problems. Mediation was tested by a 1 *df* Chi-Square change test of the model with and without estimating direct paths from witnessing community violence to conduct problems (also refer to Table 3, “Introduction” section). The model fit changed significantly after deleting the respective path: ${\text{χ}}_{\text{(19)}}^{\text{2}}$ = 152.21, *p* < 0.001; RMSEA = 0.075, CI = 0.064–0.086; CFI = 0.969; change in ${\text{χ}}_{\text{(1)}}^{\text{2}}$ = 64.46, *p* < 0.001, suggesting that reactive and proactive aggression were not acting as full mediators. In order to assess partial mediation further, the path between witnessing community violence and conduct problems was constrained to the regression weight of a direct effects model. A significant change in model fit (${\text{χ}}_{\text{(19)}}^{\text{2}}$ = 241.75; RMSEA = 0.097, CI = 0.086–0.108; CFI = 0.948; change in ${\text{χ}}_{\text{(1)}}^{\text{2}}$ = 154.00; *p* < 0.001) supported partial mediation. The association between witnessing community violence and conduct problems (i.e., the path coefficient) still remained highly significant (beta = 0.25, *p* < 0.001) even when accounting for the indirect effects of proactive and reactive aggression.

**Table 3:** Fit statistics for all models.

Measure/Model	χ^2^(df)	RMSEA	CFI
1. Mediation
*All paths free*
Overall sample
1.1 No direct path^3^	152.21 (19)	0.075 (0.064; 0.086)	0.969
1.2 Total effects^4^	87.75 (18)	0.056 (0.044; 0.067)	0.984
Change in fit 1.1–1.2	130.81 (1), *p* < 0.001		
2. Mediation
Multilevel Analysis^1^
*All paths free*
2.1 No direct path^3^	156.08 (38)	0.051 (0.043; 0.060)	0.953
2.2 Total effects^4^	129.88 (36)	0.047 (0.038; 0.056)	0.963
Change in fit 2.1–2.2	26.2 (12), *p* < 0.001		
*All primary paths equated across group*^2^
2.3 Total effects^4^	188.07 (41)	0.054 (0.046; 0.061)	0.942
Change in fit 2.3–2.2	58.19 (5), *p* < 0.001		
*Selected paths free*
2.4 Total effects^4^	130.78 (37)	0.045 (0.037; 0.053)	0.963
Change in fit 2.4–2.2	0.9 (1), *n.s.*		
*Selected paths free, insignificant paths deleted*
2.5 Total effects^4^	146.25 (51)	0.039 (0.031; 0.046)	0.962
Change in fit 2.5–2.4	15.47 (14), *n.s.*		

*^1^Refers to the two-group analysis, i.e., CD and controls analyzed separately; ^2^assumption of no group differences; ^3^full mediation model; ^4^partial mediation model*.

### Proactive and Reactive Aggression as Mediators between CVE and Conduct Problems in Adolescents with Conduct Disorder and Healthy Controls

A multilevel analysis examining the mediating effects of reactive and proactive aggression on the association between witnessing community violence and conduct problems separately within children and adolescents with CD and healthy controls and controlling for site and moderating effects of SES, age and gender revealed partly differential effects of aggression subtypes between groups.

**Figure 3:**
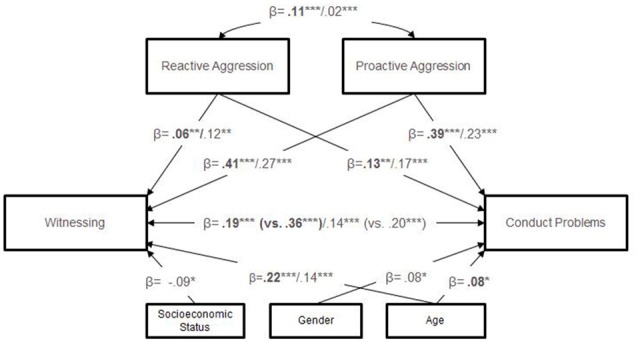
Standardized path coefficients of mediation analysis by group. *Note*: results for CD group are displayed in bold, with those for the control group presented in normal font; coefficients in brackets represent the direct path model (analyses were controlled for site); only significant coefficients are displayed. ***p < 0.001; **p < 0.01; *p < 0.05.

When examining the impact of witnessing violence, we followed a series of models to determine the best explanatory model (see “Materials and Methods” section above). A first unconstrained model fit the data well (see Table 3, “Materials and Methods” section). A constrained model significantly decreased model fit, indicating that there were at least some differences in the model paths between adolescents with CD and controls (${\text{χ}}_{\text{(41)}}^{\text{2}}$ = 188.07, *p* < 0.001; RMSEA = 0.054, CI = 0.046–0.061; CFI = 0.942; change in ${\text{χ}}_{\text{(5)}}^{\text{2}}$ = 58.19, *p* < 0.001). Re-examination of the first model and chi-square change tests confirmed significant increases in model fit when compared to the constrained model for all primary paths except for the path between reactive aggression and witnessing violence (1. path between witnessing violence and conduct problems: ${\text{χ}}_{\text{(40)}}^{\text{2}}$ = 174.27, *p* < 0.001; RMSEA = 0.052, CI = 0.044–0.060; CFI = 0.947; change in ${\text{χ}}_{\text{(1)}}^{\text{2}}$ = 13.80, *p* < 0.001; 2. path between reactive aggression and conduct problems: ${\text{X}}_{\text{(40)}}^{\text{2}}$ = 165.06, *p* < 0.001; RMSEA = 0.050, CI = 0.042–0.058; CFI = 0.951; change in ${\text{χ}}_{\text{(1)}}^{\text{2}}$ = 23.01, *p* < 0.001; 3. path between proactive aggression and conduct problems: ${\text{χ}}_{\text{(40)}}^{\text{2}}$ = 150.99, *p* < 0.001; RMSEA = 0.047, CI = 0.039–0.055; CFI = 0.956; change in ${\text{χ}}_{\text{(1)}}^{\text{2}}$ = 37.08, *p* < 0.001; 4. path between proactive aggression and witnessing violence: ${\text{χ}}_{\text{(40)}}^{\text{2}}$ = 184.18, *p* < 0.001; RMSEA = 0.054, CI = 0.046–0.062; CFI = 0.943; change in ${\text{χ}}_{\text{(1)}}^{\text{2}}$ = 3.89, *p* < 0.05). Therefore, all other paths were unconstrained in a selected-paths-free model. This select model fit the data significantly better than the constrained model (${\text{χ}}_{\text{(37)}}^{\text{2}}$ = 130.78, *p* < 0.001; RMSEA = 0.045, CI = 0.037–0.053; CFI = 0.963; change in ${\text{χ}}_{\text{(4)}}^{\text{2}}$ = 57.29, *p* < 0.001). Further, the select model did not significantly decrease model fit compared to the unconstrained model (change in ${\text{χ}}_{\text{(1)}}^{\text{2}}$ = 0.9, *n.s*.). In a final step, all non-significant paths were deleted. The fit of this final model was not significantly worse than that of the select model (change in ${\text{χ}}_{\text{(14)}}^{\text{2}}$ = 15.47, *n.s.*). Therefore, Figure 3 contains the standardized path coefficients from the final, most parsimonious model.

Across groups, the coefficients demonstrate a partial mediation effect. Further, in both groups both aggression subtypes had a significant impact on the association between witnessing violence and conduct problems, however, proactive aggression showed a stronger effect. Between groups, children and adolescents with CD showed a significantly stronger link between witnessing violence, proactive aggression and conduct problems compared to controls.

Furthermore, moderating effects of age, gender and SES were considered. Across groups, age played a significantly moderating role with regard to witnessing violence, such that older individuals tended to witness more violent events (CD: beta = 0.22, *p* < 0.001; controls: beta = 0.14, *p* < 0.001). In addition, older children and adolescents with CD showed significantly more conduct problems compared to their younger counterparts (beta = 0.08, *p* < 0.05). With regard to gender, male controls witnessed significantly more violence than female controls (beta = 0.08, *p* < 0.05). Finally, controls with a lower SES tended to show significantly more conduct problems than control subjects with a higher SES background (beta = −0.09, *p* < 0.05).

## Discussion

The present study demonstrated that recent witnessing of community violence is strongly positively associated with levels of current conduct problems in both children and adolescents with CD as well as healthy controls. Furthermore, the association between witnessing community violence and conduct problems remained significant in both groups even when including aggression subtypes (i.e., reactive/proactive aggression) as mediators in the model while controlling for the moderating effects of SES, gender and age and accounting for site effects. Proactive aggression had a stronger impact on the association between witnessing community violence and conduct problems than reactive aggression in both children and adolescents with CD and controls. When comparing the two groups, proactive aggression accounted for a greater proportion of the relationship between witnessing violence and conduct problems in children and adolescents with CD. Increased age was associated with greater rates of witnessing violence in both groups, while it was additionally associated with greater conduct problems in adolescents with CD. For controls, a lower SES was associated with greater conduct problems and being male was associated with greater exposure to witnessed violence. As such, the results of the present study are in line with findings on the well-established link between CVE and conduct problems, and extend the existing literature by demonstrating that: (1) the association between recent witnessing and current conduct problems is strongly detectable even in a group with a formal diagnosis of CD as well as a group with no clinical impairments; (2) the associations between recent witnessing of violence and current conduct problems persist even when accounting for reactive/proactive aggression across adolescents with CD and healthy controls; (3) the association between recent witnessing of violence and current conduct problems is primarily explained by proactive aggression across the two groups. Thus, the present study highlights the importance of not only taking into account early childhood risk factors known to predict the development of conduct problems and CD (Loeber et al., 2009; Bernhard et al., 2016), but also focusing on current factors in the young person’s life, such as witnessing community violence, that are likely to maintain or exacerbate conduct problems.

The present study indicates that a strong association between recent witnessing of violence and current conduct problems exists even in groups characterized by the absence of any clinically significant impairment or the presence of CD. Past studies have never investigated the relationship between CVE and conduct problems in an exclusively healthy population nor in an adolescent sample with CD. The fact that current findings indicate a robust positive association in both groups allows us to reject the possibility of an ecological fallacy due to potential confounding effects within community samples comprising a mixture of healthy and clinically impaired adolescents.

Furthermore, the present finding that recent witnessing of community violence has a strong impact on current conduct problems in children and adolescents with CD as well extends the results of studies suggesting that greater levels of violence exposure and high levels of conduct problems tend to co-occur (Sanchez et al., 2013; Cecil et al., 2014; Voisin et al., 2016). Results of a longitudinal study indicated bi-directional effects between CVE and conduct problems, suggesting a downward spiral (Mrug and Windle, 2009). The present finding of a strong association for children and adolescents with CD might point to a similar pattern. However, findings also underline that in the presence of a high rate of CVE as well as severe levels of conduct problems, eventually a ceiling effect sets in, where the strength of association between the two variables is reduced or even becomes negative. Gaylord-Harden et al. (2017) specifically investigated the cumulative impact of CVE on psychopathology in a male adolescent community sample. CVE was found to show a curvilinear relationship with internalizing problems and a positive linear relationship with violent behavior. Present findings suggest that in the case of an extremely violent group with high levels of CVE at baseline, effects of CVE on subsequent violent behavior may be much smaller due to ceiling effects.

Self-reported proactive and reactive aggression subtypes did not fully explain the link between recent witnessing of community violence and current conduct problems for children and adolescents with CD as well as for healthy controls. As such, the present findings are in line with studies indicating persisting effects of CVE on conduct problems when controlling for baseline levels of aggression (Schwab-Stone et al., 1995; Farrell and Bruce, 1997; Miller et al., 1999; McCabe et al., 2005; Weaver et al., 2008). Specifically, the finding that the association between witnessing violence and conduct problems remains when controlling for aggression subtypes suggests that it is not just the CD individual’s own level of reactive/proactive aggression that explains the link between witnessing community violence and conduct problems.

Furthermore, results of the present study showed that, for both groups, proactive aggression had a stronger mediational effect on the link between witnessing violence and conduct problems than reactive aggression. This finding aligns with studies on chronic CVE and associated desensitization processes (i.e., emotional numbing and use of aggression increasingly being seen as acceptable) resulting in higher levels of externalizing behavior (Ng-Mak et al., 2004; Boxer et al., 2008; Mrug et al., 2016; Gaylord-Harden et al., 2017). Translating these findings to the present study, one might expect that those children and adolescents witnessing more violence in their neighborhoods may have also undergone desensitization processes that have led them to become more proactively aggressive. In turn, these desensitized children and adolescents might also more readily seek out situations where violence is likely to occur or commit acts of violence which they also “witness”.

Reactive aggression, on the other hand, explained less of the association between witnessing violence and conduct problems than proactive aggression. In addition, the mediating effects of reactive aggression on the relationship between witnessing violence and conduct problems were similar in children and adolescents with CD and healthy controls. This result is surprising in the context of studies that have identified impulsivity as a correlate and predictor of CVE (Lambert et al., 2010) as well as of studies that have identified impulsivity as a relevant moderator of the effects of CVE on adolescent deviant behavior (Low and Espelage, 2014). However, Monahan et al. (2015) previously suggested that declines in impulse-control happen only in response to *increases* in CVE rates independent of an individual’s baseline rate (Monahan et al., 2015). While we have not directly investigated this, the impact of differences in CVE exposure rates over time (e.g., a change that might occur if one moves from a high to a low violence neighborhood) is something that should be considered in future studies. Furthermore, the current findings could partly be explained by the *type* of CVE assessed. Studies have shown that as the proximity of the exposure increases, the effects on emotional distress and internalizing symptoms increase as well (Fowler et al., 2009). Compared to direct victimization, witnessing community violence might evoke less emotional arousal and thus less of a response due to the more distal nature of the exposure. Individuals might feel less personally involved and are thereby less likely to act out in response.

Finally, consistent with the literature (Fowler et al., 2009), older adolescents in both groups tended to witness more violent events compared to their younger counterparts. In addition, older adolescents with CD showed more conduct problems than children. This finding aligns with studies identifying adolescence as the peak time for the majority of referrals to child and adolescent psychiatric clinics (Loeber et al., 2000) and studies that show that self-reported rates of violent offending are highest at age 16–17 years (Elliott, 1994). For controls, being male was linked to greater exposure to violence, while coming from a lower SES background was associated with greater conduct problems. Both findings have been demonstrated previously (Anderson et al., 2001; Buka et al., 2001; Javdani et al., 2014). The fact that gender and SES exerted no influence on levels of CVE and conduct problems for children and adolescents with CD might be based on the fact that the group represents a homogenous group. That is, children and adolescents with CD came from lower than average SES strata and the females within this group exhibited clinically significant levels of conduct problems. Furthermore, all children and adolescents with CD were exposed to some level of CVE and displayed some level of conduct problems by definition. As such, gender and SES would be expected to have less impact in this population compared to a more representative sample.

### Strengths and Limitations

While this study adds to our understanding of the specific associations and mediation effects regarding the link between CVE and conduct problems, it remains a snapshot of the sample’s situation at the time of assessment, i.e., a cross-sectional investigation. Hence, it is limited when it comes to exploring pathways of CVE to conduct problems as well as the long-term or cumulative effects of CVE. Consequently, it cannot shed light on whether CVE and/or reactive/proactive aggression precede the development of conduct problems or emerge as a consequence of conduct problems. Future studies using longitudinal designs will be able to shed more light on the consequences and persistence of such effects. Furthermore, it has been shown that cross-sectional approaches to mediation may result in misleading estimates (Maxwell et al., 2011). Again, it would be more valid to apply the present mediation models to data collected as part of a longitudinal study with repeated measurements of CVE, conduct problems and aggression subtypes.

Due to very low victimization rates in healthy children and adolescents, here we only focused on witnessing community violence. Fowler et al. (2009) concluded that witnessing and victimization were equally predictive of externalizing behaviors. Nevertheless, it would have been interesting to see whether reactive aggression might play a greater role in mediating the association between direct victimization and conduct problems.

In the present study, only age, gender and SES were considered as additional moderators. Aside from these variables, some additional important factors identified in previous research have been family structure, school characteristics and peer relationships (Buka et al., 2001; Chen et al., 2016). These variables present further risk or protective factors in the relationship between CVE and psychopathology but were unavailable to the authors. Future studies should therefore take these moderators into account when analyzing differences between children and adolescents with CD and healthy controls.

Finally, information obtained with regard to CVE and aggression subtypes relied on self-report data and may have been subject to social desirability effects (e.g., respondents exaggerating the extent of community violence in their neighborhoods to sound tough). Particularly with regard to CVE, past studies have found differences between informants, with parents reporting lower CVE rates for their offspring than the adolescents themselves (Kuo et al., 2000). As the present sample was mostly comprised of adolescents, it seemed safer to rely on self-report data in order to avoid potential under-reporting.

Despite these limitations, the present study is the first to systematically investigate and disentangle the effects of witnessing community violence and conduct problems in a clinical population (i.e., children and adolescents with CD) and a healthy population. As such, it is the first study to demonstrate that recent witnessing is related to current conduct problems in an exclusively healthy sample as well as in children and adolescents with CD even when taking their levels of reactive/proactive aggression into account. Further, this study is the first to illustrate that *recent* CVE is associated with the level of current conduct problems and therefore may play a role in the development or maintenance of conduct problems even for those with pre-existing externalizing behavior.

### Implications

An important implication for etiological models of the development of conduct problems is that neighborhood violence might be an important contributing factor, for healthy youths and particularly for children and adolescents with CD. From a clinical perspective, the strong association between witnessing community violence and conduct problems highlights the need for prevention and/or intervention strategies, as the relationship between the two variables is likely to intensify over time. The present results emphasize the need to consider recent CVE in addition to early risk factors. Correspondingly, it has been demonstrated that multimodal intervention programs with an additional focus on the adolescent’s environment (e.g., Multi-systemic Therapy, Multidimensional Family Therapy) are more effective in reducing conduct problems as opposed to programs that do not take an individual’s environment into account (Weisz and Kazdin, 2010; National Collaborating Centre for Mental Health, Social Care Institute for Excellence, 2013).

An important direction for future research will be to test for relationships between CVE and conduct problems in young people with CD and controls using a longitudinal design. Based on the findings of Fowler et al. (2009), we know that the strongest relationships hold between lifetime measures of externalizing behavior and lifetime CVE illustrating the cumulative impact of CVE on conduct problems over time (Fowler et al., 2009).

## Conclusion

Witnessing community violence is a highly prevalent experience for children and adolescents in Europe, and is strongly associated with the individual’s level of current conduct problems, in healthy controls as well as in children and adolescents with CD. As such, the present study is able to show a robust relationship between recent CVE and conduct problems not only in a clinically impaired sample but also in a healthy group, thereby reducing the possibility that previously reported associations between these variables were explained by an ecological fallacy. Furthermore, the present study demonstrates a strong association between recent CVE and current conduct problems, which is primarily mediated by proactive aggression. The challenge for the future lies in breaking the dangerous cycle of young people being exposed to community violence, and going on to perpetrate violence against others as a result.

## Author Contributions

CMF coordinates the FemNAT-CD FP7 research project. DD, AH, AF-R, SADB, KK, BH-D, GF, CMF, AP, MK and CS designed the study and took over site-specific coordination of the FemNAT-CD FP7 research project. LK, MP, HO, RV, LJ, KA, AB, AM, KG-M, IP, AW, JCR, RC, RHB, LG, SB, MG, GK, MAG-T, ES-P, RD, HL, ZK, ABG, AS and RS recruited subjects and collected data. LK drafted the manuscript together with CS, NV and MS. NMR, MP, SADB, KK, GF, CMF, AP and MK helped in manuscript preparation and critically reviewed the article.

## Conflict of Interest Statement

The authors declare that the research was conducted in the absence of any commercial or financial relationships that could be construed as a potential conflict of interest.

## Footnotes

^1^www.femnat-cd.eu
