# Supplementary material for: Community Violence Exposure and Conduct Problems in Children and Adolescents with Conduct Disorder and Healthy Controls
Source: Front Behav Neurosci. 2017 Nov 6;11:219. doi: 10.3389/fnbeh.2017.00219 (PMC5681536; doi:10.3389/fnbeh.2017.00219)
Supplement: Supplementary file 1 [file Table_1.docx]

Supplementary Material

**Community Violence Exposure and Conduct Problems
in Children and Adolescents with Conduct Disorder and Healthy Controls**

**Linda Kersten ^a,*^, Noortje Vriends ^a^,Martin Steppan ^a^, Nora M. Raschle ^a^, Martin Praetzlich ^a^, Helena Oldenhof ^b^, Robert Vermeiren ^b^, Lucres Jansen ^b^, Katharina Ackermann ^c^, Anka Bernhard ^c^, Anne Martinelli ^c^, Karen Gonzalez-Madruga ^d^, Ignazio Puzzo ^e^, Amy Wells ^d^, Jack C. Rogers ^f^, Roberta Clanton ^f^, Rosalind Frampton ^f^, Liam Grisley ^f^, Sarah Baumann ^g^, Malou Gundlach ^g^, Gregor Kohls ^g^, Miguel A. Gonzalez-Torres ^h^, Eva Sesma-Pardo ^h^, Roberta Dochnal ^i^, Helen Lazaratou ^j^, Zacharias Kalogerakis ^j^, Aitana Bigorra Gualba ^k^ , Areti Smaragdi ^l^, Réka Siklósi ^i^, Dimitris Dikeos ^m^, Amaia Hervas ^k^ , Aranzazu Fernández-Rivas ^h^, Stephane De Brito ^f^, Kerstin Konrad ^g^, Beate Herpertz-Dahlmann ^g^, Graeme Fairchild ^n^, Christine M. Freitag ^c^, Arne Popma ^b^, Meinhard Kieser ^o^, Christina Stadler ^a^**

**Correspondence:** Linda Kersten: [Linda.Kersten@upkbs.ch](mailto:Linda.Kersten@upkbs.ch)

# Supplementary Data

# Table 1. Percentage of endorsed frequency (within the past year) of SAHA witnessing subscale item by group

| **Witnessing item** | **Percentage of CD subjects/Controls reporting items by Frequency** | | | | |
| --- | --- | --- | --- | --- | --- |
|  | **never** | **1-2 times** | **3-5 times** | **6-9 times** | **10 or more** |
| Item 1 *(chasing)* | 55.0/87.4 | 27.7/9.9 | 10.5/2.4 | 3.1/0.3 | 3.7/0.0 |
| Item 2 *(threats)* | 44.9/83.6 | 30.7/13.2 | 12.0/2.0 | 5.0/1.1 | 7.4/0.2 |
| Item 3 *(beat up)* | 52.6/86.0 | 25.5/10.8 | 11.9/2.1 | 5.1/0.9 | 4.9/0.2 |
| Item 4 *(stabbing)* | 71.4/95.6 | 18.7/3.8 | 6.4/0.5 | 2.1/0.5 | 1.4/0.2 |
| Item 5 *(wounded)* | 62.0/90.4 | 23.6/7.5 | 8.3/1.7 | 4.1/0.2 | 1.9/0.3 |
| Item 6 *(shot)* | 86.4/99.1 | 10.5/0.3 | 2.1/0.2 | 0.8/0.3 | 0.2/0.2 |
| Item 7 *(discrimin.)* | 65.7/87.5 | 19.8/9.6 | 6.6/2.1 | 4.1/0.6 | 3.9/0.2 |
